# Genetic Circuit Design in Rhizobacteria

**DOI:** 10.34133/2022/9858049

**Published:** 2022-09-01

**Authors:** Christopher M. Dundas, José R. Dinneny

**Affiliations:** Department of Biology, Stanford University, Stanford, CA 94305, USA

## Abstract

Genetically engineered plants hold enormous promise for tackling global food security and agricultural sustainability challenges. However, construction of plant-based genetic circuitry is constrained by a lack of well-characterized genetic parts and circuit design rules. In contrast, advances in bacterial synthetic biology have yielded a wealth of sensors, actuators, and other tools that can be used to build bacterial circuitry. As root-colonizing bacteria (rhizobacteria) exert substantial influence over plant health and growth, genetic circuit design in these microorganisms can be used to indirectly engineer plants and accelerate the design-build-test-learn cycle. Here, we outline genetic parts and best practices for designing rhizobacterial circuits, with an emphasis on sensors, actuators, and chassis species that can be used to monitor/control rhizosphere and plant processes.

## 1. Introduction

Engineering interactions between plant roots and the environment is central to addressing many food, energy, and sustainability challenges. Next-generation plants with augmented root physiology or modified root architecture could be used to lower atmospheric CO_2_ levels [1, 2], improve crop resilience to biotic and climatic stresses [3–6], and increase agricultural yields to support a growing human population [7]. Plant synthetic biology approaches are poised to enable these advances, particularly through construction of genetic circuits that rewire plant metabolism and development [8, 9]. Ideally, genetic circuits constructed within plant cells could sense environmental conditions, computationally interpret external and intracellular cues, and actuate desired enzymatic and developmental phenotypes. However, forward engineering plants with these capabilities remain a substantial technical hurdle. Ongoing efforts have sought to fill knowledge gaps by uncovering design rules and building genetic part catalogs for plant-based circuits [10–13].

**Figure 1:**
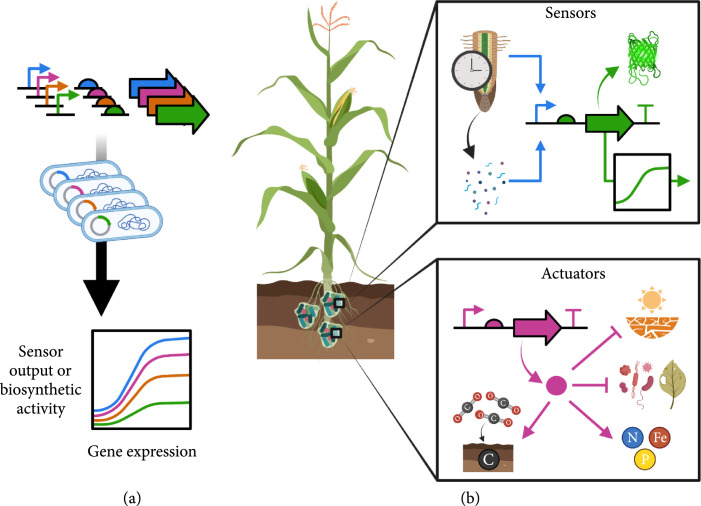
Rhizobacterial genetic circuits for monitoring and manipulating plant functioning. (a) Bacterial circuits are readily tuned at the transcriptional (promoter) and translational (ribosome binding site) level to control expression of target genes/pathways. (b) Rhizobacterial circuitry can be constructed that perceives root/environmental signals (sensors) and augments root growth/development, nutrient acquisition, stress resilience, and carbon sequestration into soils (actuators). Transcriptional sensor circuits transduce rhizosphere signals into reporter gene expression or subsequent regulation of downstream pathways. Enzymatic actuator circuits enable biosynthesis of target compounds that modulate rhizosphere chemistry or host plant physiology.

Relative to plants, genetic circuit knowledge bases are more readily available within bacterial synthetic biology [14]. Foundational concepts, such as rationally tuning gene expression [15–19], optimizing genetically encoded sensors and actuators [20–25], and implementing Boolean-type logic [26–32], are now routine tasks in bacterial systems (Figure 1(a)). Using these building blocks, genetic circuits have been built to carry out increasingly complex cellular operations, such as memory [33, 34], arithmetic [35], spatiotemporal pattern formation [36–38], and materials synthesis [39, 40].

Advances in bacterial transformation and genome engineering have greatly expanded the range of species that can host designed circuits [41–43]. This genetic tractability extends to bacteria within the plant root microbiome (termed rhizobacteria), whose members exert drastic effects on plant health, nutrient acquisition, and soil chemistry [44, 45]. Rhizobacteria effectively colonize root tissues and their growth is significantly influenced by root-exuded metabolites (e.g., carbon sources and secondary metabolites). While genetic circuitry within beneficial rhizobacteria could facilitate designer control over plant root chemistry and physiology, progress in circuit design practices and understanding of rhizosphere processes have only recently made this goal appear tangible.

Prior root microbiome engineering efforts have focused on understanding important plant-rhizobacteria interactions and isolating strains that promote plant growth in field settings. Target rhizobacteria phenotypes have included protecting plants from biotic stresses, termed biocontrol, and aiding roots in acquiring growth-limiting nutrients, such as phosphorus and nitrogen [46, 47]. More recent work has sought to understand how rhizobacteria community structure is impacted by changes in root development, environmental conditions, and species composition [48–51]. Generally, rhizobacteria research has seldom used genetic circuits beyond simple gene knockout, complementation, and overexpression constructs. Synthetic biology workflows, which have enabled design of complex sensor-computation-actuation circuits in model chassis (e.g., *Escherichia coli*) [32], remain largely unused to optimize rhizobacterial strain and community phenotypes.

Given the wealth of synthetic biology tools that can be applied towards rhizobacteria, bacterial genetic circuit design is strongly positioned to address fundamental questions in plant science and supplement plant synthetic biology in tackling challenges in agrobiotechnology. While they are yet to be field-deployed, genetic circuits could enable design of “smart” microbiomes that dynamically sense and respond to changes in plant and environmental conditions (Figure 1(b)) [52, 53]. For example, rhizobacterial circuits could be used to spatiotemporally report on changes in root-exuded metabolites that occur due to plant developmental stage or stress. These sensor inputs might trigger genetic circuitry that programs beneficial rhizobacteria colonization patterns to improve protection against competing root pathogens or abiotic stresses. Circuit-rewired rhizobacterial metabolic pathways could also tailor root chemistry to ameliorate plant nutrient limitations and increase stabilization of photoassimilated carbon within soil [54, 55].

Prerequisite to building functional rhizobacterial circuits is identifying genetic parts that target strategic root and rhizosphere processes. The purpose of this review is to highlight bacterial parts that can be applied towards this goal, including those that may be under-appreciated or unused in the context of rhizobacteria. Specifically, we discuss broad-host-range tools and target rhizobacterial chassis for designing circuits that operate in the rhizosphere. We also highlight how to use bacterial transcriptional regulators and unique reporter genes for building sensors of major root exudates and rhizosphere molecules. Finally, we summarize rhizobacterial biosynthetic actuators that yield plant growth promotion/environmental sustainability phenotypes and discuss future prospects of engineered rhizobacteria. This circuit design primer can inform plant scientists of bacterial parts and synthetic biology tools that can be used for engineering plant-rhizosphere interactions.

## 2. Tools for Building Circuits in Rhizobacteria

The wide-ranging diversity of rhizobacterial species poses a challenge when attempting to build sensor and actuator circuits that function across different strains. Genes and regulatory elements can exhibit highly varied activity within different genetic backgrounds [56, 57]. Construction of functional circuits often necessitates laborious host-specific optimization by testing libraries of promoters and ribosome binding site (RBS) sequences that fine tune gene expression [15, 18]. Plasmid-based circuits may also be challenging to port into new hosts when replication machinery is not cross-species functional [58]. Integrating circuits into host genomes can mitigate this issue, but genome engineering tools are not always available for new hosts. These constraints motivate the design of rhizobacterial circuits built with predictive tools and broad-host-range parts, as doing so can increase successful circuit construction and expedite design-build-test-learn cycles. Here, we highlight different tools that can be employed to “domesticate” rhizobacteria and permit rational assembly of genetic circuits.

### 2.1. Engineering Transcription and Translation in Rhizobacteria

Transcriptional and translational elements are critical parts for genetic circuits, but their performance can be highly variable between rhizobacterial hosts. To improve part predictability, circuit design workflows can use bioinformatic tools that aid in identifying functional promoter and RBS sequences. For example, the RBS Calculator allows users to design variable strength RBS libraries that function with high accuracy in a target host bacterium [16, 59]. The Calculator integrates sequence information from host 16S rRNA and RBS-adjacent parts to predict mRNA translation rates. This tool has enabled facile optimization of sensor and actuator circuits within diverse rhizobacteria, including *Pseudomonas*, *Klebsiella*, *Rhizobium*, and *Bacillus* spp. [59–62]. In contrast, tools for promoter identification have relied upon rhizobacterial genome mining for common promoter motifs [63, 64], as predicting promoter DNA-protein interactions is more challenging than RBS mRNA-rRNA interactions. However, recent biophysical models have made progress towards elucidating *E. coli* promoter design rules [65], and similar strategies might be capable of forward engineering promoters in rhizobacterial chassis.

Circuit predictability can also be increased using orthogonal gene expression machinery that decouples requirements from native host resources [66]. An RNA polymerase from T7 bacteriophage (T7 RNAP) is frequently used for this purpose, as it transcribes genes downstream of its cognate promoter independent of host RNAP machinery and with high activity/specificity. Notably, T7 RNAP exhibits broad-host-range functioning across the Proteobacteria and Firmicutes clades. Ryu et al. showed that T7 RNAP could serve as the primary transcriptional controller when optimizing nitrogen-fixation pathways in cereal-colonizing rhizobacteria [61]. Similarly, Wang et al. leveraged T7 RNAP to rapidly prototype fluorescently labeled strains of *Brachypodium distachyon*-colonizing *Pseudomonas simiae* [67]. Further orthogonalization of T7 RNAP/promoter variants [68, 69] and the ability to self-regulate T7 RNAP expression [70, 71] demonstrate the system’s versatility for building chassis-independent circuits in rhizobacteria. While host-decoupled translation machinery, such as orthogonal ribosomes [72–75], remains a relatively nascent technology, they might also be used to increase the chassis range of designed circuits.

### 2.2. Broad-Host-Range Plasmids

Genetic circuits are frequently assembled into plasmid DNA molecules that can be used to transform rhizobacteria. Multicopy plasmids replicate separately from the host chromosome and facilitate rapid prototyping of genetic circuits without *a priori* genome sequence information. However, rhizobacterial circuit design can be hampered by the limited host range of common plasmid origins of replication. Plasmid-based circuits that use the *E. coli*-functional ColE1 and p15A origins cannot be directly ported into most rhizobacteria [76]. Furthermore, plasmid instability in the absence of antibiotics—a scenario often encountered during plant colonization by rhizobacteria—may lead to circuit failure due to cell division-driven plasmid loss [77]. To address these limitations, rhizobacterial circuits can include genetic parts that decrease their reliance on host replication machinery and increase their maintenance in the absence of selection.

Several toolkits have been developed to ease assembly of broad-host-range plasmids. The Bee Tool Kit (BTK) [78], Bacterial Expression Vector Archive (BEVA) [79], and Standard European Vector Architecture (SEVA) [80–82] are a few prominent examples of broad-host-range plasmid systems that use modular cloning practices. These plasmids use genetic parts (i.e., regulatory elements, target genes, resistance markers, and origins of replication) flanked by distinct Type II or Type IIS restriction sites, which enables modular and hierarchical assembly of genetic designs. Notably, these systems report the characterization of broad-host-range origins of replication, such as RK2, BBR1, and RSF1010, that function within diverse plant and animal commensal bacteria. RSF1010 is particularly noteworthy, as it is one of the few known origins that replicates across Proteobacteria, Actinobacteria, and Firmicutes [83, 84]. Although antibiotic resistance is typically required for rhizobacterial plasmid maintenance, antibiotics can affect plant growth. To increase antibiotic-free maintenance, parts for plasmid stabilization can also be incorporated into broad-host-range plasmids. In the BEVA system, the RK2-derived *par* locus substantially mitigates plasmid loss by rhizobacteria grown in non-selective media [85, 86]. This system and others rely upon plasmid-encoded toxins and antitoxins, whereby plasmid loss results in a lack of antitoxin production and a promotion of toxin-mediated cell death [87, 88]. Together, these broad-host-range plasmid toolkits provide a wealth of useful parts that can be leveraged for rhizobacterial circuit design. Regardless of the cloning system chosen, circuit designers should consider standardizing their plasmid backbones with toolkit-derived origins and stability regions to increase circuit portability and efficacy in new chassis.

### 2.3. Genome Engineering Rhizobacteria

To avoid potential circuit-impairing effects of plasmids (e.g., variable cell-to-cell copy number) [89], rhizobacterial circuit designers can leverage genome engineering. In contrast to multicopy plasmids, genome-integrated circuits replicate with bacterial chromosomes in the absence of selective pressure and maintain copy numbers close to one. Early methods for genome engineering, such as plasmid-mediated homologous recombination and integrative transposons [90, 91], remain widely used to generate targeted deletions and deliver circuit cargo into rhizobacteria. More recent tools have expedited the timeframe for rhizobacterial genome engineering by possessing wider host ranges, higher genetic specificities, and multiplex editing capabilities.

Improved domestication strategies have been a critical advancement in synthetic biology and enabled circuit design in many previously intractable rhizobacteria. Brophy et al. miniaturized the integrative and conjugative elements from *Bacillus subtilis* (mini-ICE*Bs*1) to develop a *B. subtilis* donor strain that conjugates circuits into a wide range of gram-positive recipients [41]. This system integrates circuits at a conserved leucine tRNA locus and was successfully used to transfer inducible GFP expression and nitrogen-fixation circuits into soil isolates and mixed communities. Wang et al. developed a chassis-independent recombinase-assisted genome engineering (CRAGE) tool that domesticates diverse Proteobacteria and Actinobacteria using a randomly integrating transposon [42]. Without requiring *a priori* genome sequence information, the conjugated transposon chromosomally integrates Cre recombinase and an antibiotic resistance gene flanked by mutually exclusive *lox* sites. This permits delivery of additional *lox*-flanked genetic cargo in subsequent rounds of conjugation and antibiotic selection. This system was capable of functioning in rhizobacteria and delivering CRISPR cargo to target host biosynthetic pathways [67, 92]. Recently, CRISPR-transposase technology was used to perform species- and site-specific genome editing within soil bacteria communities [43]. CRISPR-transposases facilitate gRNA-targeted integration of cargo to desired chromosomal loci [93–95]. The Doudna and Banfield labs used this platform to perform gene knockout and deliver carbon utilization pathways to community-residing species in a selection-free environment [43]. These next-generation genome engineering tools are poised to enable circuit assembly across new chassis within the rhizosphere, including many unculturable rhizobacteria.

## 3. Selecting Rhizobacterial Chassis for Genetic Circuits

Circuit functioning within rhizobacteria is underpinned by the ability of these chassis to effectively colonize root tissues. Circuits used to monitor and modulate plant growth would ideally be deployed in strains exhibiting high extents of colonization (i.e., bacterial cells per root). Additionally, the inputs and outputs of rhizobacterial circuits are strongly tied to root colonization patterns. For example, rhizobacterial sensors that preferentially localize to different root compartments (e.g., epiphytes vs. endophytes) and anatomical regions (e.g., root hairs vs. root tips) will be exposed to distinct soil nutrient, exudate, and developmental stimuli [96, 97]. Colonization patterns will similarly affect bacterially augmented nutrient uptake and phytohormone signaling. Furthermore, the ecological stability of rhizobacteria within microbial communities can impact their extent of colonization and the success of implemented circuitry [98, 99]. Thus, in addition to the parts used to build rhizobacterial circuits, colonization capabilities of the host chassis should be considered equally important design parameters.

### 3.1. Native and Engineered Colonization Activity

Rhizobacterial chassis can be selected based on known colonization and strain characteristics. Strains that localize to distinct root regions, such as elongation zone-colonizing *Bacillus* [100], could be used to target circuitry to specific developmental stages. Other root-colonizing strains may be chosen due to existing plant growth-promoting phenotypes. For example, *Pseudomonas* and rhizobial species frequently serve as testbeds for engineered circuitry due to their well-studied root colonization behavior and functioning in biocontrol and nitrogen fixation [101, 102]. While many other bacteria possess advantageous genetic backgrounds (e.g., biosynthetic gene clusters), poorer rhizosphere persistence by these strains motivates genetic optimization of colonization. This can be accomplished by targeting genes that control colonization-relevant traits, such as chemotaxis, root attachment, biofilm formation, and plant immune system evasion [103]. Optimized strains might increase production of biofilm-promoting exopolysaccharides [104, 105] or express immune system-evading flagellin monomer proteases [106]. Alternatively, experimental evolution strategies could be used to select for high-colonizing strains that persist in the rhizosphere over multiple inoculation/selection cycles. This approach has evolved *Pseudomonas* and *Bacillus* species with elevated root colonization through mutations in genes encoding global regulators, biofilm development, and motility machinery [107–110]. Refining genetic design rules for root colonization should further our ability to spatiotemporally control circuit functioning and transfer colonization phenotypes into potentially beneficial bacteria that do not natively colonize plants.

### 3.2. Chassis-Microbiome Interactions

Bacterial fitness and interactions within rhizosphere communities are also important design constraints when choosing a circuit-hosting chassis. While an engineered strain may be an effective root colonizer in monoculture, it could be outcompeted by other bacteria in a community setting. This would concomitantly dilute circuit-driven sensing and plant growth-promoting phenotypes. Biocontrol strain chassis (e.g., *Pseudomonas protegens* and *Bacillus velezensis*) may mitigate this issue [111, 112], as these strains produce a spectrum of antimicrobial compounds that aid their establishment within the root microbiome. Conversely, rhizobacterial chassis may negatively impact rhizosphere community structure and encourage proliferation of bacteria that cause adverse plant phenotypes. Selection of keystone rhizobacterial species as chassis might stabilize community effects on plant hosts by mediating critical interactions across microbial taxa [113]. *Variovorax* spp. are a recent example of this, as they broadly suppress root growth inhibition in complex communities through a genus-conserved auxin degradation operon [51]. Likewise, chassis strains should be evaluated for their ability to elicit microbiome-modulating plant disease phenotypes, such as induced systemic resistance or induced systemic susceptibility [114–117]. Engineered circuitry might also alter chassis composition within communities through targeted killing of population members [118] or signal-based regulation of population density [119].

## 4. Sensors for Root Exudates and Rhizosphere Compounds

Rhizobacteria encounter diverse chemical stimuli within roots and in the rhizosphere. Root exudates include many primary (e.g., sugars, organic acids, and amino acids) and secondary metabolites (e.g., phytohormones), whose concentrations continuously vary based on their location of release from the root, plant developmental stage, environmental conditions, and in response to biotic/abiotic stresses [45, 120]. Additionally, the rhizosphere contains many inorganic plant nutrients (e.g., nitrate and phosphate) that also affect root microbiome activities. The intimate relationship between these molecules and plant functioning makes them attractive sensing targets for monitoring plant health and actuating interventions when necessary. Accordingly, rhizobacterial genetic circuits could be leveraged to spatiotemporally perceive and report on the concentration of key rhizosphere compounds.

**Figure 2:**
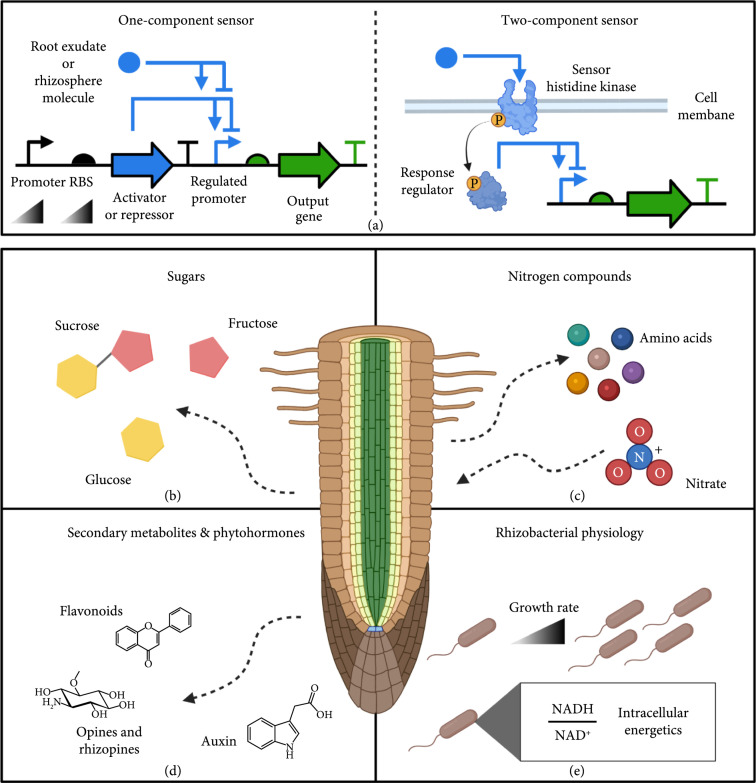
Rhizobacterial sensor circuit design. (a) Sensor circuits control output gene expression using small molecule-responsive regulators that alter transcription at their cognate promoters. One-component sensors encode a single allosteric transcription factor that either activates or represses transcription upon binding the target root/rhizosphere molecules. Two-component sensors contain a transmembrane histidine kinase, whose extracytoplasmic domain binds to target molecules to allosterically alter its kinase activity. The phosphorylation activity of the histidine kinase subsequently alters the transcription factor activity of the response regulator at its regulated promoter. Performance of both sensor circuit types can be tuned by altering expression of sensor/regulator proteins at the promoter and RBS level. Sensor circuits can be built to detect root exudates and rhizosphere molecules such as (b) sugars, (c) nitrogen compounds, and (d) secondary metabolites and phytohormones. (e) Sensors can also be used to detect general rhizobacterial processes that are affected by root colonization and exudation.

While many genetic parts can be used to create biosensors, small molecule-responsive transcriptional regulators possess several advantages for rhizobacterial sensor design. Transcriptional sensor circuits typically function by expressing one- or two-component regulator proteins that modulate expression of cognate promoters placed upstream of a regulated gene (e.g., a reporter protein) (Figure 2(a)) [121, 122]. These circuits have well-established design rules and exhibit predictable patterns of activity based on gene expression (e.g., the Hill equation) [14, 123, 124]. Modeled response functions relate inducer molecule concentrations to output activity, which are parameterized by their sensitivity (i.e., threshold inducer concentration) and dynamic range (i.e., ratio of maximal induction to uninduced state). These parameters are engineered by changing the promoter and RBS strength of both regulator and output genes. Protein engineering can also tune response functions by modulating the regulator’s ligand/DNA affinity or kinase/phosphatase activity [22, 125, 126]. Furthermore, outputs of transcriptional sensors can be connected to additional downstream modules, such as multi-input logic gates and actuator elements.

Here, we characterize various transcriptional regulators that can be used to build rhizobacterial sensor circuits for major root exudates and rhizosphere compounds (Table 1). We also discuss transcriptional sensors that can monitor root-induced changes in overall bacterial physiology (e.g., growth rate). Lastly, we identify non-fluorescent sensor outputs that can potentially monitor rhizobacteria activity in complex plant-soil systems.

**Table 1:** Genetic parts for constructing rhizobacterial sensor circuits.

Sensor target	Sensor gene(s)-output promoter^a^	Detection range	Bacterial chassis-tested plant/environment	Reference
Sucrose	*scrR* - P*_scrY_*	10 *μ*M to 1000 *μ*M	*Erwinia herbicola* - wild oat (*Avena barbata*)	[130, 134]
Fructose	n/a - P*_fruB_*	1.67 *μ*M to 167 *μ*M	*Erwinia herbicola* - common bean (*Phaseolus vulgaris*)	[135]
Glucose	n/a - P_gluA7_	500 *μ*M to 10 mM	*Escherichia coli* - n/a	[139]
Glucose-6-phosphate	*uhpABC* - P*_uhpT_*	0.5 *μ*M to 4 *μ*M	*Escherichia coli* - n/a	[138]
Nitrate	*fnr* and *narXL* - P*_narG_*	0.1 *μ*M to 10 mM	*Enterobacter cloacae* - wild oat (*Avena fatua*)	[144]
Nitrate	*narX* and *narL/ydfI* - P_*yd*fJ115_	5.62 *μ*M to 562 *μ*M	*Bacillus subtilis* - fertilized soil	[126]
L-Tryptophan	n/a - P*_aatl_*	0.1 *μ*M to 100 *μ*M	*Erwinia herbicola* - wild oat (*Avena barbata*)	[130]
L-Phenylalanine	n/a - P*_phhA_*	10 *μ*M to 5000 *μ*M	*Rhizobium leguminosarum* - pea (*Pisum sativum*)	[136]
L-Proline	*putR* - P*_putA_*	0.1 *μ*M to 500 *μ*M	*Rhizobium leguminosarum* - pea (*Pisum sativum*)	[156]
L-Lysine	*lysG* - P*_lysE_*	5 mM to 25 mM	*Corynebacterium glutamicum* - n/a	[157]
L-Methionine	*lrp* - P*_brnF_*	0.2 mM to 23.5 mM	*Corynebacterium glutamicum* - n/a	[158]
L-Glutamate	*aauSR* - P*_rpoN_*	20 mM^b^	*Pseudomonas aeruginosa* - n/a	[159]
Naringenin	*fdeR* - P*_fdeA_*	2 *μ*M to 100 *μ*M	*Pseudomonas protegens* - n/a	[61, 161]
Quercetin	*qdoR* - P*_qdoI_*	10 *μ*M to 100 *μ*M	*Escherichia coli* - n/a	[161]
Luteolin	*fdeR/nodD1* - P*_fdeA(R)_*	7 *μ*M to 56 *μ*M	*Herbaspirillum seropedicae* - n/a	[162]
Apigenin	*fdeR* - P*_fdeA(R)_*	7 *μ*M to 60 *μ*M	*Herbaspirillum seropedicae* - n/a	[162]
Salicylic acid	*nahR* - P_sal_	1 *μ*M to 100 *μ*M	*Azotobacter caulinodans* - n/a	[61]
Indole-3-acetic acid	*iacR* - P*_iacH_*	1 *μ*M to 200 *μ*M	*Enterobacter soli* - n/a	[180]
2-Phenylacetic acid	*paaXK* - P*_paaA_*	10 *μ*M to 200 *μ*M	*Escherichia coli* - n/a	[185]
Octopine	*occR* - P_occ_	1 *μ*M to 10 *μ*M	*Azotobacter caulinodans* - n/a	[61]
Nopaline	*nocR* - P_noc_	100 *μ*M to 1000 *μ*M	*Azotobacter caulinodans* - n/a	[61]
*Scyllo*-inosamine	*mocR* - P*_mocB_*	1 *μ*M to 1000 *μ*M	*Rhizobium leguminosarum* - alfalfa (*Medicago sativa*), barrelclover (*Medicago truncatula*), and barley	[167]
Intracellular ribosomal biosynthesis	n/a - P*_EcrrnB_*	0.17 h^-1^ to 0.28 h^-1^	*Pseudomonas putida* - barley	[192]
Intracellular NADH/NAD^+^ ratio	*rex(Bs)* - P_ropIP_	0.38 to 0.88^c^	*Escherichia coli* - n/a	[198]

a. Sensor genes and promoters were either used directly in the cited reference or inferred. b. Only a single L-glutamate concentration was tested for sensor induction relative to its absence. c. Sensor NADH/NAD^+^ range was inferred from biochemically measured values.

### 4.1. Sugars

Sugars, such as sucrose, glucose, and fructose, are primary components of soluble root exudates (up to 65% by mass) [127, 128]. Root-exuded sugars serve as carbon sources for rhizobacteria and can be reabsorbed by roots to fulfill plant biomass and energy requirements. Sucrose is particularly noteworthy, as it is the primary carbon fixation product of photosynthesis and serves as currency in the plant’s carbon economy [129]. Based on source-sink limitations within plant tissues, sucrose is differentially transported to non-photosynthetic organs (e.g., roots) to support their growth. Concentrations of root-exuded sucrose have shown to spatially increase towards growing root tips and are significantly higher during the seedling stage of plant development [130–132]. Given this ability to spatiotemporally map root development and potentially serve as a biomarker for various plant stresses (e.g., pathogen infection), sucrose and its associated sugars appear as valuable inputs for building rhizobacterial genetic circuits (Figure 2(b)).

Sugars are common circuit inducers across bacterial synthetic biology with two of the most popular inducers being the plant-released sugars, xylose and arabinose [20, 133]. However, only a few studies from the early 2000s report constructing sucrose-inducible circuits. These sucrose sensors were engineered in the plant epiphyte, *Erwinia herbicola*, using the regulator-promoter pair from *Salmonella typhimurium*, ScrR-P*_scrY_*, and outputting reporter genes encoding either GFP, LacZ, or InaZ [130, 134]. The sucrose sensor’s detection range of 10 to 1000 *μ*M enabled detection of sucrose exuded along soil-grown *Avena barbata* roots, with a measured concentration of ca. 100 *μ*M near the root tip. Circuits can also be used to detect the release of sucrose catabolic products, glucose and fructose [135]. Pini et al. analyzed the transcriptomics profile of *Rhizobium leguminosarum* to pinpoint native promoters that upregulate genes during sucrose and fructose supplementation [136]. Biosensors were built by transforming *R. leguminosarum* with plasmids carrying identified promoters placed upstream of the *luxCDABE* operon. Quantification of *lux*-driven luminescence revealed the importance of rhizobia nitrogen fixation for high plant exudation of sucrose and fructose in pea root nodules. Modern synthetic biology optimizations could potentially upgrade these rhizobacterial circuits to increase linearity in their detection range [35] and portability to strain backgrounds lacking sensor-interfering sugar catabolism [137]. Circuits not previously tested in rhizobacteria might also be useful for sugar detection, such as other one-component/two-component circuits or those that monitor glucose-6-phosphate levels [138, 139].

### 4.2. Nitrogen Compounds

Nitrogen is a growth-limiting plant nutrient that is taken up from soils by roots and transported to aboveground organs [140]. Consequently, nitrogen compounds within the rhizosphere and those released as root exudates are closely tied to plant and microbiome functioning [120]. While it is not an appreciable root exudate, nitrate is a major plant nitrogen source and is exogenously supplemented through chemical fertilization of soils. Relative to other forms of nitrogen, root exudation of sugars appears to be heavily downregulated by nitrate supplementation [141]. This suggests strong ties between nitrate levels and the feeding of resident bacteria growing on roots [132]. In addition to its plant and microbial assimilation, nitrate serves as a dissimilatory respiratory source for denitrifying bacteria under anaerobic conditions [142]. Nitrate uptake processes are also linked to changes in root developmental signaling, with lower rhizosphere nitrate levels triggering a foraging response of auxin-promoted lateral root growth [143]. Taken together, construction of rhizobacterial nitrate sensors could aid precision agriculture-guided fertilization, improve understanding of nitrogen effects on rhizosphere communities, and potentially forecast changes in root development (Figure 2(c)).

Nitrate-sensing circuits can be built that function in the soil environment. DeAngelis et al. developed a plasmid-based sensor using the promoter of the *Escherichia coli* nitrate reductase gene, *narG*, placed upstream of either the *gfp* or *inaZ* reporter genes [144]. Under anaerobic conditions, P*_narG_* is induced by nitrate and the global regulator protein FNR. Since FNR is inactive under aerobic conditions, they also co-expressed an oxygen-insensitive mutant of FNR (FNR-L28H) [145], which permitted aerobic nitrate inducibility in the range of 0.1 *μ*M to 10 mM. When testing soil-grown grass *Avena fatua* colonized by *Enterobacter cloacae* carrying this sensor, it was observed that reporter activity was highest in nitrate-amended bulk soils and substantially lower around root tissue [144]. This was interpreted as rapid assimilation of nitrate by roots, with reporter-based rhizosphere concentration estimates at 1 *μ*M. A limitation of this sensor was that it presumably relied upon the chassis’ native expression of the sensor histidine kinase-response regulator pair, NarX-NarL, to modulate P*_narG_* activity [146, 147]. In contrast, the Tabor Lab built a nitrate sensor circuit by directly inserting *Escherichia coli narX*-*narL* into the genome of *Bacillus subtilis* [126]. To mitigate heterologous expression issues with the *E. coli nar* promoter, the C-terminal of NarL was replaced with an orthologous DNA binding domain from *B. subtilis* YdfI, which enabled nitrate-inducible *gfp* expression from the *Bacillus* functional P_*yd*fJ115_ promoter. Protein engineering to decrease NarX phosphatase activity increased sensor sensitivity and dynamic range, relative to circuits with the wild type NarX. Using their optimized sensor, they demonstrated nitrate detection in chemically fertilized soils over a range of 5.62 to 562 *μ*M. While their biosensor was not used in a plant root environment, a recent report testing a similar NarX-NarL sensor in mouse-colonizing *E. coli* Nissle 1917 suggests promise for *in situ* sensing of nitrate in microbiomes [148].

Amino acids are another important form of rhizosphere nitrogen. These small molecules are naturally abundant in soils and can serve as organic plant nitrogen sources, particularly during inorganic nitrogen deficiency [149, 150]. Like nitrate, amino acids strongly interact with plant signaling pathways that modulate root architecture [151]. In contrast to inorganic forms of nitrogen, amino acids constitute a large fraction of root exudates. Amino acids readily nourish root microbiota and are significant chemoattractants for rhizobacteria [152, 153]. Plants dynamically alter their amino acid exudation during growth and under various environmental contexts, including nutrient deficiencies [128], elevated atmospheric CO_2_ levels [154], and exposure to microbial products [155]. Given these roles, amino acids may be useful inducers for rhizobacterial circuits (Figure 2(c)).

The diversity of proteinogenic amino acids gives rise to numerous genetic parts for their quantitation in the rhizosphere. Jaeger et al. built a tryptophan biosensor by fusing the *inaZ* reporter gene to the tryptophan aminotransferase gene, *aatl*, within the genome of epiphyte *E. herbicola* [130]. The *aatl* promoter is induced in the presence of tryptophan, which enabled the biosensor strain to detect higher levels of the amino acid around emerging lateral roots of soil-grown *Avena barbata*. Similarly, Pini et al. used the native phenylalanine-inducible promoter of phenylalanine-4-hydroxylase from *R. leguminosarum* to trigger *luxCDABE*-based luminescence within this bacterium [136]. Plasmid-based expression of the biosensor revealed temporal dynamics of phenylalanine exudation within the rhizosphere and root nodules of pea. The same group built another genetic sensor in *R. leguminosarum* for proline—a particularly abundant root exudate—using the native *putR* regulator gene and a promoter region proximal to a proline catabolism gene, *putA* [156]. Previously characterized genetic parts from non-rhizobacteria might also be co-opted to build rhizobacterial sensors for exuded amino acids, such as lysine, methionine, and glutamate [157–159].

### 4.3. Secondary Metabolite and Phytohormones

In addition to primary metabolites, plants exude several secondary metabolites and hormones that shape plant development, rhizosphere chemistry, and microbial activities. For many of these specialized compounds, genetic parts can be leveraged to build bacterial sensor circuits (Figure 2(d)). One example of this is with flavonoids: a class of plant-released compounds that regulate various plant-microbe symbioses [160]. Sensor circuits were built in bacterial chassis, including rhizobacteria, to detect the flavonoids naringenin, quercetin, luteolin, and apigenin [61, 161, 162]. Del Valle et al. showed that flavonoid biosensors can be used to analyze flavonoid-containing soil, which enabled the researchers to study how soil organic matter affects naringenin bioavailability and subsequent root nodulation frequency with *Medicago sativa* [163]. Given the role of flavonoids in diverse health and consumer products, flavonoid-sensing bacterial circuits might be used to aid plant metabolic engineering efforts [164].

Engineered exudation of secondary metabolites can facilitate designer interactions between plants and rhizobacteria. Towards this goal, transgenic plants were created that express biosynthetic pathways for non-native signaling molecules, opines and rhizopines [165–167]. These compounds are naturally produced by *Agrobacterium*-infected plants and root nodulating rhizobia, respectively, and serve as determinants of bacterial colonization and catabolism [168]. Plants with engineered opine exudation exhibited increased colonization by opine-catabolizing bacteria [165, 166] and can potentially be paired with biosensors for octopine and nopaline [61]. The Poole Lab demonstrated that root exudation of the orthogonal rhizopine, *scyllo*-inosamine, could mitigate bacterial catabolism and enable specific transkingdom signaling by bacteria carrying optimized rhizopine sensors [167]. Development of further orthogonal signaling circuitry will be critical for multiplexing communication to individual rhizobacteria and engineering communities whose members actuate distinct tasks [169].

Bacterial circuits can also be built to sense plant hormones. For example, salicylic acid is a pathogen defense-related hormone and has been the target for sensor circuits built in rhizobacterial chassis [61, 136, 170]. Auxins are particularly important hormones, due to their significant effects on plant growth [171] and their biosynthesis/catabolism by rhizobacteria [172, 173]. While genetic methods for auxin detection are frequently used in eukaryotic systems [174–176], there are surprisingly few examples of auxin-inducible circuits engineered in bacteria. Indole-3-acetic acid (IAA) is one of the primary auxins within plants [177], but there appears to be no bacterial biosensor circuits explicitly built for detecting this molecule. Nonetheless, studies on bacterial IAA degradation pathways have identified IAA-responsive transcriptional regulators [178–180]. Although their use in circuits has not been tested, these parts are promising candidates for IAA sensor design. Additionally, Wang et al. created biosensors for non-IAA indole compounds and proposed their engineered regulator could be adapted for IAA specificity [181]. Bacterial circuits have also been built to sense the less understood auxin, 2-phenylacetic acid (PAA). PAA similarly affects plant growth and rhizobacterial activities [182, 183], albeit at higher concentrations than IAA. Bacterial circuits were optimized to output the fluorescent reporters, GFP and RFP, in response to PAA and its related compounds, 4-hydroxyphenylacetic acid, and 2-phenylethylamine [184, 185]. Future developments in hormone sensing circuitry might enable design of rhizobacterial strains that rewire plant signaling to influence plant growth and immunity [186].

### 4.4. Rhizobacterial Physiology

Root exudates affect general physiological processes of rhizobacteria, such as cell growth rate and intracellular metabolite levels. As root exudate composition varies during plant growth, rhizobacteria adjust their metabolism to feed on different sets of encountered nutrients [132]. Sensor circuits could be leveraged to spatiotemporally interrogate these generalized exudation effects and program downstream circuit responses by rhizobacteria (Figure 2(e)).

Bacterial growth rates have long been correlated with cellular ribosomal RNA (rRNA) levels and activity from rRNA promoters [187–189]. These phenomena are explained by translation-based growth limitations—bacteria need more ribosomes to grow faster. Thus, reporter genes placed downstream of rRNA promoters can potentially act as cell growth rate and metabolic activity sensors [190, 191]. Using this approach, the activity of *Pseudomonas* on barley roots was studied by chromosomally integrating a *rrnB* ribosomal promoter from *Escherichia coli* upstream of an unstable *gfp* variant [192]. Cells from growth rate-controlled chemostats exhibited ribosomal content and GFP fluorescence that were linearly related to growth rates in the range of 0.17 to 0.28 h^-1^ (i.e., doubling times of ca. 4 to 2.5 h). When analyzing this strain’s growth on barley seedling root tips, epifluorescence microscopy revealed highest GFP levels at the edges of microcolonies formed on border cells. This contrasted another barley-colonizing *P. putida* strain that constitutively expressed GFP via the *lac* promoter, where broad cellular fluorescence was observed across the entire root tip. In a separate study, rRNA-driven expression of unstable GFP in *Pseudomonas fluorescens* similarly showed higher fluorescence at the root tips of colonized alfalfa [193]. Strains that expressed more stable GFP variants yielded fluorescence throughout the entire root system. These results support increased exudation rates at growing root tips and suggest that rRNA promoters can be used to map spatial gradients of bacterial activity on roots. Although rRNA-bacterial activity correlations may break down under certain growth rates and environmental conditions [194], these genetic elements provide an immediately obvious part to test for building growth-responsive rhizobacterial circuits. Understanding of rRNA promoter functioning within rhizobacterial hosts may be further improved through circuit-physiology modeling [195].

Intracellular bacterial energetics is also tied to root exudation. The intracellular ratio of global energy carriers, such as NAD^+^/NADH, changes based on carbon source availability and has been tied to rhizobacterial colonization [196]. While not tested in rhizobacteria, the Rex regulator can be used to build transcriptional circuits that detect NAD^+^/NADH ratios [197, 198]. Liu et al. showed that the Rex regulator from *B. subtilis*, B-Rex, can induce fluorescent reporter expression under conditions of high intracellular NADH (i.e., anaerobic growth) by derepressing a promoter that contains a Rex operator. Porting this energetics sensor into rhizobacteria could potentially improve understanding of carbon use efficiency with root exudates [199] and complement other sensors that interrogate influential rhizosphere parameters (e.g., oxygen levels) [200, 201].

### 4.5. Reporters for Monitoring Plant-Bacteria Interactions in Soil

Fluorescent reporters have been powerful tools for unraveling plant-rhizobacteria interactions but are challenging to use in soil settings. Imaging of rhizobacterial fluorescence typically requires disruptive preparation of root samples [202], which can potentially perturb colonization patterns of soil-grown plants. Although live fluorescent imaging can be accomplished for plants grown in optically transparent soil [203, 204], differences in chemical and physical properties of these synthetic systems may alter rhizobacterial colonization and plant growth when compared to natural soils. To address these limitations and build genetic circuits that report on rhizosphere dynamics *in situ*, reporters that function within soil environments are needed.

Luminescence has been used as an output for many rhizobacterial genetic circuits, and in some situations can be used in soil environments. Many of these circuits leverage the *lux* operon, which biosynthesizes enzymes and metabolite substrates required for bacterial luminescence [205]. Rellán-Álvarez et al. built a rhizotron imaging system capable of phenotyping luminescent plants and rhizobacteria in soil, termed Growth and Luminescence Observatory for Roots (GLO-Roots) [206]. The thin dimensions of their rhizotrons enabled cameras to image the entire root system of transgenic *Arabidopsis thaliana*, *Brachypodium distachyon*, and *Setaria viridis* that expressed luciferase genes and were watered with luciferin substrates [207]. They also showed that this system could image a root-colonizing *P. fluorescens* strain that constitutively expressed *lux*. Considering recent robotics and automation upgrades to their system (GLO-Bot) [208], this rhizotron platform could potentially be used to prototype sensor circuits in a soil environment.

Non-optical reporters have been used to build genetically encoded sensors for soil bacteria processes. The Silberg Lab developed a soil-functional reporter system using sensor circuits that biosynthesized indicator gaseous compounds, methyl bromide and ethylene [163, 209–211]. In their initial circuits, a ratiometric gas signal was generated by inducer-regulated methyl halide transferase (MHT) expression/methyl bromide production and constitutive ethylene forming enzyme (EFE) expression/ethylene production. Sensor strains were mixed into soil within sealed containers, and headspace concentrations of the output gasses were quantified by mass spectrometry. These sensors generated typical Hill function responses and could detect quorum sensing molecules within soil matrices modulated by rhizobacteria, *Bacillus thuringiensis* and *R. leguminosarum*. Although non-ethylene ratiometric gasses would be needed to mitigate interference with native plant signaling, generation of methyl halide alone can permit functional sensor activity [163, 211]. Additionally, their gas reporter approach appears compatible with existing infrastructure used to study plant release/uptake of gaseous compounds [212]. Ultrasound technology for imaging soil-grown plant roots [213] might similarly be adapted to detect acoustic reporters (i.e., gas-filled protein nanostructures) produced by rhizosphere bacteria [214]. While functioning of these reporters has yet to be demonstrated in rhizobacterial species, they have shown promise for *in situ* reporting within the mammalian microbiome [215].

## 5. Rhizobacterial Actuators

**Figure 3:**
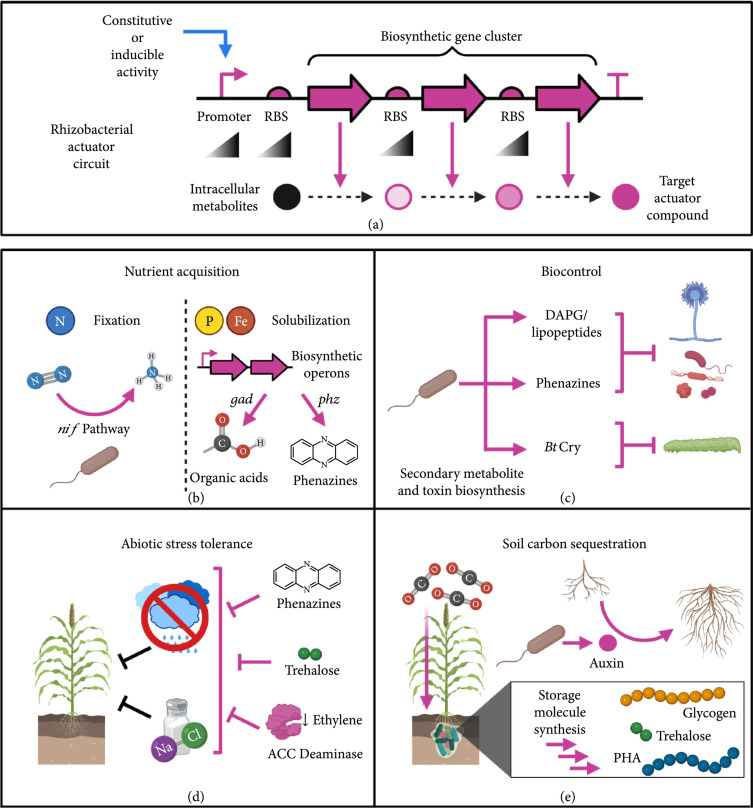
Rhizobacterial actuator circuit design. (a) Actuator circuits encode biosynthetic pathways for small molecules or proteins that affect root/rhizosphere processes. Biosynthetic genes are often assembled into operons or clusters to enable shared regulation of related functions (e.g., metabolic enzymes and transport proteins). Biosynthetic activity can be tuned by altering the strength of constitutive/inducible promoters for gene clusters or the RBS strength of individual genes. (b) Nutrient acquisition is a common plant growth-promoting actuation where rhizobacterial genes enable nitrogen fixation and phosphate/iron solubilization. (c) Rhizobacterial-biosynthesized compounds can facilitate biocontrol of fungi, bacteria, and insects that detrimentally affect plant health and yields. (d) Bacterial synthesis of target molecules and enzymes can modulate rhizosphere/root chemistry to increase plant resilience to drought and salinity stresses. (e) Rhizobacterial conversion of root exudates into storage molecules and stimulation of root growth can increase the residence time of plant-fixed carbon in soils to aid sequestration efforts.

Actuator elements of engineered rhizobacteria drive agriculture- and sustainability-relevant phenotypes in colonized plants. In rhizobacterial circuits, these genes traditionally encode individual proteins or biosynthetic pathways that control nutrient acquisition, biotic stress resilience, and plant growth promotion (Table 2). More recently, rhizobacterial actuators have been proposed to address effects of climate change by improving plant resilience to abiotic stresses and augmenting carbon sequestration into soil. To maximize rhizobacterial effectiveness in these applications, circuit designers would benefit from knowing what actuators can be used, how they can be optimized, and how they interface with broader ecological and environmental processes (Figure 3(a)). In this section, we will highlight recent progress in these areas and emerging genes/pathways that can be leveraged for actuator engineering.

**Table 2:** Genetic parts for constructing rhizobacterial actuator circuits.

Actuation(s)	Biosynthesized compounds and actuator gene(s)	Regulation and notes	Bacterial chassis-host plant	Reference
Nitrogen fixation	Refactored *nif* cluster from *Klebsiella oxytoca*	IPTG- and aTc-inducible T7 RNAP controller regulated subdivided *nif* operons	*Klebsiella oxytoca* - n/a	[60]
Nitrogen fixation	62 member library of combinatorially assembled *nif* clusters from *Klebsiella oxytoca*	Variable strength RBSs for each *nif* gene and an IPTG-inducible T7 RNAP controller for subdivided *nif* operons	*Klebsiella oxytoca*, *Escherichia coli* - n/a	[220]
Nitrogen fixation	12 native and engineered *nif* clusters from diverse bacterial orders (Enterobacterales, Pseudomondales, Rhizobiales, Rhodobacterales, Rhodospirillales, Bacillales, and Oscillatoriales)	Native regulation; variable strength RBSs for each *nif* gene and an IPTG-inducible T7 RNAP that controlled expression of the *nif* master regulator, NifA	*Azorhizobium caulinodans*, *Rhizobium* sp., *Pseudomonas protegens* - n/a	[61]
Nitrogen fixation	Native *nif* cluster with engineered postranslational regulation that increases ammonia secretion	Engineered unidirectional adenyltransferase (uAT) inhibits glutamine synthetase	*Azospirillum brasilense* - *Setaria viridis*	[221]
Nitrogen fixation	Native *nif* cluster with engineered postranslational regulation that increases ammonia secretion	Multiple copies of engineered uAT expressed to increase genetic redundancy and prolong BNF activity	*Azospirillum brasilense* - *Setaria viridis*, maize (*Zea mays*)	[222]
Phosphate/iron solubilization	Citric acid produced by expressing citrate synthase (*gltA*) and citrate transporter (*citC*)	Constitutive *lac* promoter drove genome-integrated citrate operon	*Pseudomonas fluorescens*, *Pseudomonas protegens* - n/a	[228]
Phosphate/iron solubilization	2-Ketogluconic acid produced by expressing of gluconate dehydrogenase operon (*gad*)	IPTG-inducible *tac* promoter and native *gad* operon promoter	*Enterobacter asburiae* - n/a	[230]
Phosphate/iron solubilization	Gluconic acid and 2-ketogluconic acid produced by expressing PQQ cofactor synthesis gene cluster (*pqq*) and *gad* operon	Constitutive *lac* promoter	*Herbaspirillum seropedicae* - rice (*Oryza sativa*)	[229]
Phosphate solubilization	82 phytases from diverse bacteria were refactored for *Proteobacteria* expression and tested in 3 species to create 185-phytase expressing strains	IPTG-inducible	*Pseudomonas putida*, *Pseudomonas simiae*, *Ralstonia* sp. - *Arabidopsis thaliana*	[233]
Biocontrol	2,4-DAPG produced by heterologously expressing *phlACBDE* biosynthetic locus	Native regulation	*Pseudomonas fluorescens* - wheat, tomato	[253]
Biocontrol	2,4-DAPG is overproduced by altering transcription of native *phlACBD* operon	Genomic deletion of Pseudomonas sigma regulator gene (*psrA*) relieves *phlACBD* transcriptional repression	*Pseudomonas fluorescens* - n/a	[254]
Biocontrol	2,4-DAPG is produced by heterologously expressing *phlDACB* gene cluster	Constitutive *lac* promoter	*Pseudomonas* sp. - rice, sorghum, wheat	[255]
Biocontrol	Iturin A lipopeptide overproduced by engineering promoter of native *itu* operon	Constitutive strong promoter (C2up) place upstream of *itu* operon	*Bacillus amyloliquefaciens* - n/a	[256]
Phosphate/iron solubilization, biocontrol, abiotic stress resilience	Phenazines overproduced by engineering the 5’ UTR of the native *phz* operon	Deletion of a 90 bp region in the 5’ UTR of *phz* operon relieved transcriptional and translational repression	*Pseudomonas chlororaphis* - wheat	[258, 271]
Phosphate/iron solubilization, biocontrol, abiotic stress resilience	Phenazine-1-carboxamide (PCN) overproduced by engineering promoters of native *phz2* gene cluster and glutamine aminotransferase (*phzH*)	Native promoters of *phz2* and *phzH* gene clusters replaced with strong quorum sensing- and thermo-regulated P*_rhlI_*	*Pseudomonas aeruginosa* - n/a	[259]
Phosphate/iron solubilization, biocontrol, abiotic stress resilience	Phenazine *N*-oxides overproduced by heterologously expressing phenazine *N*-monooxygenase (*naphzNO1*) and monooxygenases (*phzS* or *phzO*)	Native P*_phz_*, P*_phzS_*, and P*_phzO_* promoters used to drive *naphZNO1*-*phz*, *phzS*, and *phzO* gene clusters, respectively	*Pseudomonas chlororaphis* - n/a	[261]
Phosphate/iron solubilization, biocontrol, abiotic stress resilience	PCN overproduced by replacing chromsomal *phzO* gene with *phzH*	*phzH* regulated by native promoter of *phzO*; several negative regulators of *phz* biosynthesis machinery and metabolite pool were deleted from genome (*lon*, *rsmE*, *psrA*, *rpeA*, *parS*, *pykF*)	*Pseudomonas chlororaphis* - n/a	[260]
Phosphate/iron solubilization, biocontrol, abiotic stress resilience	Phenazine-1,6-dicarboxylic acid (PDC) overproduced in 5 chassis hosts using 4 refactored PDC gene clusters that contain *phzABG* or homologs	IPTG-inducible T7 RNAP controlled PDC gene cluster expression	*Pseudomonas simiae*, *Aeromonas salmonicida*, *Escherichia coli*, *Dickeya solani*, *Xenorhabdus doucetiae* - n/a	[268]
Biocontrol	*Bacillus thuringiensis* Cry toxin evolved to bind cabbage looper (*Trichoplusia ni*) cadherin-like receptor not bound by wild-type toxin	Protein evolved using phage-assisted continuous evolution (PACE) over more than 500 generations	*Escherichia coli* - n/a	[265]
Abiotic stress resilience/soil carbon sequestration	Elevated trehalose production by overexpressing trehalose-6-phosphate synthase (*otsA*)	Constitutive *lac* promoter	*Rhizobium etli* - common bean (*Phaseolus vulgaris*)	[276]
Abiotic stress resilience/soil carbon sequestration	Elevated trehalose production by heterologously expressing *ReotsA* or chimeric fusion of trehalose-6-phosphate synthase/trehalose-6-phosphate phosphatease (*BIF*) from *Saccharomyces cerevisiae*	Constitutive *lac* promoter	*Azospirillum brasilense* - maize (*Zea mays*)	[277]
Abiotic stress resilience/soil carbon sequestration	Elevated trehalose production by heterologously expressing *otsA* and trehalose-6-phosphate phosphatase (*otsB*)	Constitutive *lac* promoter	*Pseudomonas putida* - green pepper (*Capsicum annum*)	[278]
Abiotic stress resilience/soil carbon sequestration	Elevated trehalose production by overexpressing maltooligosyltrehalose synthase (*treY*) and maltooligosyltrehalose trehalohydrolase (*treZ*)	IPTG-inducible *tac* promoter	*Corynebacterium glutamicum* - n/a	[311]
Abiotic stress resilience	Plant ethylene levels lowered by heterologously expressing a surface displayed ACC deaminase (*inaK*-N/*acdS*)	Strong constitutive promoter from *Enterobacter cloacae* (fragment 132a/HQ834306)	*Enterobacter* sp., *Kosakonia* sp. - rice	[289]
Abiotic stress resilience	Ethylene biosynthesis by heterologously ethylene forming enzyme (*efe*)	Strong constitutive promoter (P_1_)	*Escherichia coli*, *Shewanella oneidensis*, *Bacillus thuringiensis* - n/a	[209]
Soil carbon sequestration	Poly(3-hydroxybutyrate-co-4-hydroxybutyrate) (P(3HB)) produced by expressing (P(3HB)) synthesis operon (*phaCAB*) and succinate degradation pathway (*orfZ*, *4hbD*, *sucD*)	Native promoters used for *phaCAB* and *orfZ*, strong constitutive promoter (P*_pdc_*) used for *4hbD-sucD*	*Escherichia coli* - n/a	[306]
Soil carbon sequestration	Glycogen biosynthesis from native *glg* operon	Native regulation, induced by raffinose/trehalose and repressed by glucose	*Lactobacillus acidophilus* - n/a	[310]
Soil carbon sequestration	Indole-3-acetic acid biosynthesis by expressing 2-tryptophan monooxygenase (*iaaM*) and indole-3-acetamide hydrolase (*iaaH*)	Quorum sensing autoinducer production (*luxI*) regulates *iaaMH* placed downstream of P*_luxI_*	*Cupriavidus pinatubonensis* - *Arabidopsis thaliana*	[318]

### 5.1. Nitrogen Acquisition

Rhizobacteria can aid root acquisition of growth-limiting nutrients and potentially eliminate agricultural requirements for chemical fertilizers. Owing to the high energy and economic costs associated with synthetic nitrogen fertilizer [216], biological nitrogen fixation (BNF) has been a primary actuation targeted by rhizobacterial engineers [217]. BNF pathways are natively found in rhizobia that mutualistically colonize legume root nodules [218]. Although these pathways enable substantial delivery of fixed nitrogen (e.g., ammonium and amino acids) to legumes, rhizobial BNF activity is heavily restricted to within legume root nodules. This has motivated circuit designers to transfer nitrogen fixation (*nif*) pathways into bacteria that reside outside root nodules (e.g., free-living bacteria), as these engineered strains could deliver nitrogen to nodule-lacking crops, such as cereal grasses (Figure 3(b)) [219].

BNF circuit design has proven challenging due to limited BNF activity in different host backgrounds, heavy pathway regulation by environmental factors (e.g., NH_3_^+^ and O_2_ inhibition), and the large multigene footprint of *nif* pathways (11 kbp to 64 kbp). To decouple native *nif* regulation, key regulator genes (e.g., *nifA*) have been placed under the control of inducible orthogonal regulators, such as T7 RNAP. These synthetic controllers, coupled with entire pathway refactoring (i.e., reordering genetic elements to remove native regulation and enable designer control over individual gene expression), have optimized transcription and translation of *nif* pathways for new host chassis [60, 61, 220]. Recently, Schnabel and Sattely improved bacterial BNF activity by engineering a posttranslational step that regulates ammonia release from diazotrophic *Azospirillum brasilense* [221]. Under typical BNF conditions, glutamine synthetase (GS) siphons away generated ammonia to produce bioassimiliated glutamine. Since GS activity is regulated by a bidirectional adenyltransferase (AT), the group engineered a unidirectional adenyltransferase (uAT) that keeps GS inhibited and could elevate bacterial ammonia delivery to *Setaria viridis*. This system was further optimized by expressing multiple copies of uAT, which buffered against evolutionary instability from ammonia overproduction [222]. Genetic redundancy might also be applied with *nif* pathway components to prolong BNF activity in new hosts, as metabolic burden by the pathway (nitrogenase can account for 20% of cell mass) increases likelihood of circuit breakage.

### 5.2. Phosphate and Iron Solubilization

Rhizobacterial actuators can also assist plants in extracting key nutrients from soil. While phosphate and iron are terrestrially abundant, they are typically found within mineral precipitates or highly adsorbed to mineral surfaces [223, 224]. This limits their soil solubility and accessibility by plants, which necessitates exogenous application in agriculture. To decrease reliance on synthetic fertilizer and mitigate its environmental runoff effects, nutrient-solubilizing rhizobacteria have been used to promote phosphate and iron mobilization around roots (Figure 3(b)).

Many rhizobacterial chassis natively produce high amounts of organic acids and siderophores, which facilitate release of mineral-bound nutrients for plant uptake [225–227]. Metabolic engineering in rhizobacteria can increase nutrient solubilization through expression of pathways that elevate secretion of citric acid, gluconic acid, and 2-keto-D-gluconic acid [228–230]. Bacterial siderophore biosynthesis can also be genetically optimized [231, 232], but this has yet to be demonstrated in a rhizobacterial context. In a separate approach, Shulse et al. showed that circuits expressing 82 diverse phytases in *P. putida*, *P. simiae*, and *Ralstonia* sp. can enzymatically liberate P_i_ from organic phosphate (phytate) and increase its uptake by *Arabidopsis* [233]. Antibiotic metabolites produced by *Pseudomonas* spp., termed phenazines, can also increase Fe uptake by plants through reductive dissolution of minerals [234, 235]. Recently, McRose and Newman demonstrated that phenazines are capable of liberating mineral-bound phosphate within natural soils [236]. Given their proposed role as “keystone metabolites” of soil community structure [237, 238] and amenability to metabolic engineering [239], phenazine-producing circuits could be a promising means to improve iron/phosphate uptake by plants and augment existing phenazine pools in agricultural soils [240–242].

### 5.3. Biotic Stress Resilience

Biocontrol is an important tool in agricultural pest/disease management and a major focus of rhizobacterial engineering [46, 243]. Biocontrol strains protect plants from insect herbivory and fungal- and bacterial-borne diseases, which minimizes the requirement for costly and environmentally impactful chemical pesticides. This is primarily accomplished through bacterial biosynthesis of biocontrol compounds, such as toxic proteins and secondary metabolites [244]. To optimize biocontrol activity and increase the host range of protected plants, biocontrol circuits can be engineered in native biocontrol strains or new rhizobacterial chassis (Figure 3(c)).

Genome mining in rhizobacteria has pinpointed many genetic determinants of biocontrol activity, with early progress focusing on model biocontrol strains from the *Pseudomonas* and *Bacillus* genera. *Pseudomonas protegens* Pf-5 was found to protect diverse plants (e.g., cotton, wheat, cucumber, and tomatoes) against soil-borne fungal/bacterial pathogens by possessing biosynthetic gene clusters for iron-chelating siderophores (e.g., pyoverdine and pyochelin), antifungals (e.g., 2,4-diacetylphloroglucinol/DAPG), and hydrogen cyanide [111, 245–248]. Similarly, *Bacillus velezensis* FZB42 was shown to protect potato, wheat, and lettuce by encoding several antimicrobial polyketides (e.g., bacillaene and macrolactin) and antifungal lipopeptides (e.g., fengycin and bacillomycin D) [249–252]. Identification of these pathways has enabled metabolic engineering approaches to overproduce target compounds within rhizobacteria [253–256].

Antibiotic phenazines from *Pseudomonas* spp. have also served as biocontrol circuit outputs. These compounds are broadly produced by rhizosphere-dwelling pseudomonads and are thought to actuate microbial killing through ROS generation [257]. Increasing *Pseudomonas* phenazine titers is possible by engineering promoter and 5′ UTR elements of *phz* biosynthetic pathways [258, 259]. Alternatively, combining *phz* components from different *Pseudomonas* spp. can tailor production towards phenazine derivatives with higher antibacterial activity, such as phenazine-1-carboxamide and phenazine N-oxide [260, 261].

Resistance to biocontrol agents is a mounting agricultural problem and has motivated identification of new insecticidal, antifungal, and antibiotic actuators. An example of this is with *Bacillus thuringiensis* (*Bt*): an important biocontrol agent that kills insect larvae by Cry/Cyt protein toxins [262]. These toxins bind to insect receptor proteins, which leads to formation of lethal pores in their cell membranes. Although *Bt* toxins are widely used in pest management, their field effectiveness has waned as insects acquire *Bt* resistance [263, 264]. Since *Bt* toxins are proteins, their insecticidal activity and susceptibility to resistance can potentially be optimized through protein engineering. To test this hypothesis, Badran et al. used a continuous evolution strategy to engineer Cry variants that bind a cabbage looper (*Trichoplusia ni*) receptor protein untargeted by the wild-type toxin [265]. Evolved toxins were 335-fold more potent against wild-type resistant insects and could possibly be expressed in new *Bt* strains. Advancements in high throughput sequencing, genetic engineering, and metabolite analysis methods have similarly increased the rate of discovery for novel biocontrol actuators [266, 267]. Wang et al. powerfully demonstrated this by using CRAGE to activate nine gene clusters from insect pathogens, *Photorhabdus* and *Xenorhabdus*, when screening for insecticidal metabolites [42]. Rapid genome engineering of 25 diverse Proteobacteria hosts, including a few rhizobacteria, allowed them to identify pathways that produced previously elusive metabolites. This same group used CRAGE to rapidly assay phenazine biosynthetic gene clusters and identify those that facilitate high titers of the bioactive phenazine derivative, phenazine-1,6-dicarboxylic acid [268]. While it is likely inevitable that individual biocontrol actuators will become obsolete as their targets acquire resistance, the modularity of rhizobacterial chassis and biosynthetic circuitry should enable replacement actuations to be rapidly deployed.

### 5.4. Abiotic Stress Resilience

Climate change-induced stresses, such as drought and high salinity, are becoming increasingly problematic within agricultural regions. These stresses devastate crop health and yields and are predicted to impact 50% of all arable land by 2050 [269]. In addition to other plant growth-promoting effects, rhizobacteria have demonstrated significant potential for improving plant resiliency to abiotic stresses. Bacteria can ameliorate stress through a combination of biosynthesized protective compounds, biofilm formation within the rhizosphere, and stress-priming modulation of root chemistry [270]. Despite the complex polygenic nature of stress protection traits, key genetic contributors have been identified in rhizobacterial species. As few genetic circuits have been built for the express purpose of abiotic stress protection, understanding these factors can clue us into useful genetic parts and circuit design rules (Figure 3(d)).

Adding to their multipurpose functionality, phenazines from *Pseudomonas* spp. have demonstrated an ability to improve plant drought and salinity tolerance. Mahmoudi et al. found that wheat colonized by phenazine-producing *Pseudomonas chlororaphis* 30-84 strains exhibited higher relative water content and improved survivability after periods of water deficit, relative to wheat colonized by a phenazine-null mutant and uninoculated controls [271]. Improved drought tolerance might have resulted from adapted root development, as phenazine production stimulated belowground growth and increased total root length, root surface area, and the number of root tips. The same group used these *P. chlororaphis* strains to assay phenazine effects on salinity stress with wheat and similarly observed that phenazine production generally lowered salt-induced ROS accumulation [272]. Although the exact mechanism for phenazine-based protection remains unclear, it is proposed that they directly impact plant functioning (e.g., inducing stress-response pathways), indirectly assist plants by modifying the rhizosphere environment (e.g., increasing bacterial abundance/biofilm formation), or both. These results suggest that existing phenazine biosynthesis circuits could be repurposed as actuators for drought and salinity tolerance within rhizobacterial chassis [259–261].

Trehalose biosynthesis is another rhizobacterial actuation that confers colonized plants with drought and osmotic stress tolerance. Trehalose is a disaccharide composed of two glucose monomers that naturally accumulates under osmotic stress conditions in many bacteria, animals, and plants [273]. In addition to its protective capabilities, trehalose and its derivatives are important signaling molecules that regulate plant tissue sugar levels [274, 275]. Although rhizobacteria natively produce trehalose, its overaccumulation can be engineered to improve plant stress protection. Suarez et al. demonstrated that increasing trehalose biosynthesis in *Rhizobium etli* substantially improves the growth of common bean (*Phaseolus vulgaris*) during water deficit [276]. Constitutive plasmid-based expression of the *R. etli* trehalose-6-phosphate synthase (TPS) gene, *otsA*, increased plant survival and yields by over 50% relative to those inoculated with wild-type strains. Similarly, *A. brasilense* engineered to express a chimeric fusion of trehalose-6-phosphate synthase and trehalose-6-phosphate phosphatase (TPP) from *Saccharomyces cerevisiae* was able to increase survivability and root length of maize subjected to drought [277]. In another study, trehalose overaccumulation in *P. putida* was engineered by expressing *otsA* (TPS) and *otsB* (TPP) from desiccation-tolerant strain *Microbacterium* sp. 3J1 [278]. Under 14 days of no watering, this strain increased the dry weight and relative water content of green pepper up to 1.2-fold relative to wild-type inoculated plants. Like other biosynthesized protectant compounds, it remains unclear whether trehalose directly provides osmoprotection to plant cells or indirectly confers these benefits [279]. For example, rhizobacteria might be triggering trehalose-linked adaptive stress response pathways within plants [280].

Rhizobacterial circuits that improve biofilm formation might physiochemically buffer roots from abiotic stresses. Biofilm components, such as exopolysaccharides, can directly hydrate the rhizosphere and reduce movement of harmful Na^+^ ions from soils to roots [281–283]. Drought tolerance of *A. thaliana* colonized by *Bacillus amyloliquefaciens* was shown to be dependent on biofilm formation genes, such as *epsC* and *tasA* [105, 284]. Biofilm-deficient mutants yielded plants with substantially lower survivability, biomass, and root development. Since many of these strains also exhibited impaired root colonization, biofilms could also indirectly influence cell density-dependent interactions with *Arabidopsis*, such as bacterial modulation of stress signaling pathways [105]. For example, many rhizobacteria promote plant growth under drought and salinity stress by disrupting ethylene signaling [285]. Ethylene is an important phytohormone that generally inhibits root growth and has shown to accumulate in plant tissues during abiotic stress [286]. Rhizobacteria that natively or heterologously express ACC deaminase, an enzyme that degrades the precursor to ethylene (ACC), can lower plant ethylene levels and lessen ethylene-mediated inhibition of growth [287, 288]. To improve activity of the typically intracellularly localized ACC deaminase, Liu et al. engineered a surface displayed variant of the *acdS* gene by translationally fusing it to the InaK membrane anchoring motif [289]. Expressing this variant in endophytic *Enterobacter* and *Kosakonia* increased germination rates up to 3-fold for colonized rice under high salinity (2.5% NaCl), relative to wild-type strains. Since ethylene can also positively impact plant salinity responses [290, 291], rhizobacterial stress protection circuits might leverage combinations of ethylene production [209] and ACC degradation to tune ethylene levels depending on plant species and environmental conditions.

### 5.5. Soil Carbon Sequestration

Increasing plant-mediated transfer of atmospheric carbon into soils can combat anthropogenic CO_2_ emissions and reverse the effects of climate change [292]. Soils are the largest terrestrial reservoir of carbon (ca. 3-fold more carbon in soils than the atmosphere) and receive photosynthetic inputs from root growth and exudation [293]. Root-derived carbon enters soil through the rhizosphere, where rhizobacterial communities play a significant role in stabilizing soil organic matter (SOM) pools [294, 295]. Rhizobacteria either assimilate carbon inputs into soil-stabilized biomass or respire these compounds as CO_2_ that is released back into the atmosphere. Elevating SOM levels is generally accomplished by increasing the formation of stable root and rhizobacterial biomass. This might occur through higher root growth/exudation and formation of carbon-rich root/bacterial biopolymers. Since rhizobacteria can actuate both these processes with biosynthetic pathways, rhizobacterial circuit design could be employed to optimize soil carbon sequestration (Figure 3(e)).

Despite significant variability in carbon use efficiencies and CO_2_ losses across microbial species [296, 297], microbe-derived biomass constitutes an enormous portion of soil carbon pools (up to 50% is composed of dead microbial cells) [298, 299]. Rhizobacterial circuits might further augment this balance by increasing the conversion of root exudates into biomass components with high soil residence times. For instance, many soil bacteria naturally generate long-lived storage molecules that serve as mobilizable energy reservoirs and protectants against environmental stresses [300]. As these storage compounds can make up to ca. 25% of cell dry mass, overexpressing relevant biosynthetic pathways in rhizobacterial chassis could enable creation of high SOM-forming strains. One target for biosynthesis may be polyhydroxyalkanoates (PHA), which are energy storage polyesters of many soil bacteria [301]. PHA exhibits a wide range of molecular structures and can improve starvation endurance of nitrogen-fixing root colonizers, such as *A. brasilense* and *Sinorhizobium meliloti* [302–305]. Primary metabolism intermediates (e.g., acetyl-CoA) are converted into PHA polymers by expressing the *pha* or *phb* gene clusters, with PHA synthase (*phaC*) exerting high control over the specificity of polymerized PHA monomers [306–308]. Glycogen is another high energy polymer that is catabolized by rhizobacteria under stress conditions [309]. Given the abundance of glycogen monomers in root exudates (i.e., glucose) [127], rhizobacteria may be ideal chassis for its hyperaccumulation by overexpressing the *glg* biosynthetic pathway [310]. While trehalose is a relatively small polymer (2-mer), it also serves as a bacterial storage molecule that could be produced at high titers by engineered circuitry [311]. Since biosynthesis of any storage molecule will likely impact rhizobacterial growth and root colonization capabilities, it will be critical to evaluate if engineered strains actuate a net increase of SOM in field settings.

Plant-based sequestration efforts have largely focused on optimizing crop root characteristics that deposit more carbon belowground [1, 2]. Carbon-storing ideotypes include crops with elevated root system biomass and deeper/steeper root architectures [312]. Modeling analyses predict that even a 2-fold increase in these traits on 90% of United States cropland could offset up to 60% of the country’s transportation emissions [313]. As a complement to plant engineering approaches, engineered rhizobacteria could actuate desired root phenotypes through targeted biosynthesis of phytohormones, such as auxin. Within root cells, the concentration of auxin/IAA regulates expression of genes that control cell division, differentiation, and elongation [314, 315]. Rhizobacteria that locally modulate these concentrations can alter overall features of root development [316, 317]. For example, Zúñiga et al. showed that a quorum sensing-based positive feedback circuit within *Cupriavidus pinatubonensis* could induce biosynthesis of IAA and increase primary root length and lateral root number of colonized *A. thaliana* [318]. Future circuits that target IAA biosynthesis to specific root regions might allow bacteria to more precisely control the design of root architectures [186].

Phytohormone biosynthesizing rhizobacteria could also be used to tailor root chemical compositions towards higher levels of long-lasting biomolecules. Lignin and suberin are proposed carbon sequestration targets [319], as these biopolymer components of root cell walls and nutrient diffusion barriers exhibit high recalcitrance to microbial/abiotic degradation [320, 321]. Despite their biotic stability in soils, their abundance within roots is substantially affected by rhizobacteria [322, 323]. Salas-González et al. demonstrated that, relative to axenic plant growth, bacterial colonization generally decreases endodermal suberization of *A. thaliana* roots by inhibiting endodermal abscisic acid (ABA) signaling. This suggests that rhizobacteria engineered to biosynthesize ABA could reverse signaling inhibition and increase suberin deposition within root tissues. While ABA biosynthesis has been observed by rhizobacterial isolates [324–326], further understanding on the genetic basis of this actuation is required to design ABA-producing circuitry.

## 6. Challenges to Deploying Rhizobacterial Genetic Circuits

Although rhizobacterial genetic circuits have the potential to augment growth and functioning of agriculturally relevant plants, key technical, regulatory, and ethical challenges will need to be considered to apply circuit-containing bacteria outside the laboratory. As with any synthetic biology endeavor, rhizobacterial circuits will require genetic optimization for the specific bacterial strain and range of environmental conditions in which they are deployed. Sensory and biosynthetic pathways (e.g., nitrogen fixation) can perform poorly in non-native bacteria due to pathway refactoring-based loss of cryptic, yet important, regulation [60, 61]. Additionally, findings from engineered rhizobacteria prototyped in monoassociation with axenically grown plants may not directly translate to field experiments; circuit-carrying strains applied to environmental soil-grown plants will likely encounter diverse microbial communities that may alter their colonization dynamics and/or perform similar/competing functions (e.g., nutrient mobilization and phytohormone modulation) [327, 328]. These challenges underscore the need to build robust genetic toolkits in non-model rhizobacteria to facilitate learn-by-design optimization of individual pathways and strains. Improving genetic tractability across chassis with varying colonization patterns can expand the ability of circuit designers to direct pathway activity to developmentally and physiologically relevant regions of the root system, such as meristematic or high nutrient flux zones [329, 330]. Since maintaining bioinoculant colonization in the complex and competitive microbiome of plants remains a major hurdle [331], access to new chassis might also increase the persistence of deployed strains. This could occur by porting genetic circuitry into dominant bacterial taxa of target rhizospheres [48, 49, 332]. While all these approaches will require rigorous assessment in field conditions, they might enable engineered rhizobacteria to outcompete native microbiota and perform beneficial plant growth-modulating activity beyond what is naturally occurring.

Perhaps the foremost issue facing application of rhizobacterial circuitry is the limited public and political acceptance of deploying transgenic bacteria in the field, which has led to strict regulation over their release [333, 334]. While field implementation of transgenic plants is similarly constrained [335], genetically modified bacteria have elicited greater fears of strain escape from deployment sites (i.e., target crops) and horizontal gene transfer to environmental microbes. This has translated to protracted and expensive regulatory barriers, which many in the scientific community view as excessive given the posed risks [336, 337]. As transgenes are an inevitable necessity for building next-generation sensor-actuator rhizobacterial circuitry, it will be important to integrate input from government regulators, bioethicists, and ecologists to study the economic/environmental benefits and risks of engineered microbes and advise on the safe, responsible, and efficacious implementation of well-characterized rhizobacterial technologies [338, 339]. Public unease regarding horizontal gene transfer/strain escape might be ameliorated by engineering strains with biocontainment circuitry [340, 341] and modeling the environmental impact of strain/transgene deployment [99, 342, 343]. Biocontainment of transgenic strains might involve designing auxotrophies that require plant-produced metabolites for bacterial growth [344, 345] or utilizing environmentally triggered kill switches [346–348]. Alternatively, strains with recoded genomes/nonstandard amino acid usage could render escaped transgenes non-functional outside the engineered host context [349–351].

Despite these formidable challenges, the ability of bacterial circuits to perform value-added biochemistries has led to increased commercialization of rhizobacterial synthetic biology for agriculture [352]. Many companies have been founded with aims to engineer rhizobacteria for plant nutrient acquisition and stress tolerance [353]. For example, Pivot Bio recently reported a free-living nitrogen-fixing *Klebsiella* strain that can increase maize yields in field trials up to 3%, relative to untreated fields [354]. Although genome editing optimized nitrogen fixation by this strain, non-transgenic status was retained through deletion of native regulatory components that decouple *nif* expression from exogenous nitrogen presence/absence. As more sophisticated engineering endeavors continue to navigate regulatory landscapes, rhizobacterial circuitry can still find utility within industrial research settings to uncover commercial opportunities afforded by bacterial manipulation of root chemistry and growth. Incorporating genetic engineering design-build-test-learn cycles into biofertilizer product development can pinpoint desirable traits of non-modified bacteria that could be bioprospected from the environment and further tested in pre-commercialization phases (i.e., field trials) [355].

## 7. Conclusions

The last two decades have led to substantial progress in bacterial synthetic biology and our understanding of plant-bacteria interactions [329, 356]. Although advances in each field were generally independent of the other, tools for optimizing genetic circuit design and domesticating non-model chassis are now positioned to accelerate engineering of rhizosphere processes. Many of the separate sensor and actuator parts we describe could be connected to dynamically report and respond to changes in plant physiology. Modern rhizobacterial circuits might look like the recent attempts to engineer nitrogen-fixing *nif* pathways that are modularly induced by plant-released compounds, such as (rhizo)pines and salicylic acid [61, 357]. One could envision similar circuits that use root exudates (e.g., sucrose and phytohormones) to trigger actuations based on plant developmental stage and surrounding community composition [132]. To connect these sensing and actuation functions, it will be necessary to port previously built transcriptional logic gate machinery into new rhizobacterial chassis [358].

As advances are made in other bacterial fields, it is tempting to speculate how these innovations might translate into new rhizobacterial sensors and actuators. The intersection of synthetic biology with materials science is one burgeoning area, where functionalized living materials are fabricated by engineered bacteria [359, 360]. Circuit-biosynthesized materials, such as amyloid-based hydrogels and cellulosic biomass [361, 362], might provide a unique means to functionalize colonized root tissues and buffer plants against environmental stresses, such as drought. Materials formed by CO_2_-fixing rhizobacteria could similarly progress climate change mitigation by augmenting carbon capture into soils [25, 363]. Additionally, rhizobacterial circuitry could be co-opted to interact with pollutants and deleterious materials that plants encounter in the environment [364]. Bacterial pathways that degrade plastics and detoxify hazardous metals could be anchored within soil by root systems as robust remediation schemes [365–368].

The next wave of synthetic biology is primed to utilize plant-associating hosts for genetic circuit design. While this review focused on designing circuits that function within rhizobacteria, rhizosphere-colonizing fungi (i.e., mycorrhizae) could also be genetically engineered to perform similar biological functions [369]. Their high prevalence in the rhizosphere [370] and mediation of key plant-environment interactions (e.g., nutrient acquisition and soil carbon storage) [371, 372] make mycorrhizae attractive engineering targets and should motivate increased genetic toolkit development. Alternatively, circuit design strategies could be adopted to engineer bacteria that colonize aboveground plant tissue compartments, known as the phyllosphere [373, 374]. Phyllosphere sensor-actuator circuits present their own unique opportunities for optimizing plant health and plant-environment interactions, such as enabling airborne pollutant remediation (i.e., phylloremediation) [375, 376]. Furthermore, plants themselves could be engineered to manipulate rhizobacterial circuits and functioning. For example, plant-produced transkingdom signaling molecules (e.g., rhizopines) were shown to trigger rhizobacterial sensor circuits and actuations [167, 357]. Alternatively, root exudates could be engineered to nutritionally tailor microbiota composition [377]. While it remains to be tested, root production of exotic carbon sources (e.g., algal polysaccharides) might give certain bacteria a growth advantage if these strains express carbohydrate-active enzymes necessary for metabolization [378]. This approach enabled engineered *Bacteroides* engraftment within the mouse gut microbiome [379] and could similarly be applied to improve strain persistence within the rhizosphere. These and other highlighted strategies demonstrate how genetic circuits can improve plant-bacteria interactions for agricultural and environmental sustainability. As engineered rhizobacteria move from the wet bench to the field, it will be exciting to see how plant-monitoring and -controlling circuits perform at scale.
